# Decoding the Formation of New Semantics: MVPA Investigation of Rapid Neocortical Plasticity during Associative Encoding through Fast Mapping

**DOI:** 10.1155/2015/804385

**Published:** 2015-07-16

**Authors:** Tali Atir-Sharon, Asaf Gilboa, Hananel Hazan, Ester Koilis, Larry M. Manevitz

**Affiliations:** ^1^Psychology Department, University of Haifa, 3498838 Haifa, Israel; ^2^Psychology Department, Yezreel Valley College, 19300 Yezreel Valley, Israel; ^3^The Rotman Research Institute at Baycrest, Toronto, ON, Canada M6A 2E1; ^4^The Centre for Stroke Recovery, Toronto, ON, Canada M6A 2E1; ^5^Psychology Department, University of Toronto, Toronto, ON, Canada M5S 3G3; ^6^Computer Science Department, University of Haifa, 3498838 Haifa, Israel

## Abstract

Neocortical structures typically only support slow acquisition of declarative memory; however, learning through fast mapping may facilitate rapid learning-induced cortical plasticity and hippocampal-independent integration of novel associations into existing semantic networks. During fast mapping the meaning of new words and concepts is inferred, and durable novel associations are incidentally formed, a process thought to support early childhood's exuberant learning. The anterior temporal lobe, a cortical semantic memory hub, may critically support such learning. We investigated encoding of semantic associations through fast mapping using fMRI and multivoxel pattern analysis. Subsequent memory performance following fast mapping was more efficiently predicted using anterior temporal lobe than hippocampal voxels, while standard explicit encoding was best predicted by hippocampal activity. Searchlight algorithms revealed additional activity patterns that predicted successful fast mapping semantic learning located in lateral occipitotemporal and parietotemporal neocortex and ventrolateral prefrontal cortex. By contrast, successful explicit encoding could be classified by activity in medial and dorsolateral prefrontal and parahippocampal cortices. We propose that fast mapping promotes incidental rapid integration of new associations into existing neocortical semantic networks by activating related, nonoverlapping conceptual knowledge. In healthy adults, this is better captured by unique anterior and lateral temporal lobe activity patterns, while hippocampal involvement is less predictive of this kind of learning.

## 1. Introduction

Current theories of declarative memory, drawing on the canonical memory systems framework, suggest two complementary memory systems [1–3]: a hippocampal-based system that specializes in rapid acquisition of specific events (episodic memory) and a neocortical system that slowly learns through statistical regularities (semantic memory). According to these theories, semantic memory is represented by neocortical structures but is acquired only through a slow consolidation [1–5] or transformation [6] processes. The initial acquisition of declarative memories (semantic or episodic) critically depends on the hippocampal memory system, which continues to support them until slow consolidation processes allow neocortical networks to represent memory independently. Contrary to this view, recent exciting findings from rats [7, 8] suggest that when new information is associated with previously known, well-integrated, knowledge (schema [9–11]) rapid neocortical consolidation occurs. Similarly, we recently reported rapid acquisition of arbitrary associations through a mechanism dubbed fast mapping (FM) in amnesic patients due to medial temporal lobe (MTL) damage [12, 13] but see [14, 15]. If true, this suggests that under some conditions the neocortex is capable of rapid leaning-induced plasticity independently of the hippocampus or with minimal hippocampal support [14].

FM was first described by Carey and Bartlett [16]. It is a process by which children infer by exclusion the meaning of new words and which supports subsequent memory for these novel associations even after a single exposure. In their experiment, Carey and Bratlett showed 3-4-year-old children two trays, one of them being red and the other olive and asked the children to bring “the chromium tray, not the red one, the chromium one.” The children retrieved the olive tray, correctly inferring that the word “chromium” refers to this unknown color; moreover, when children were asked after a week to select “the chromium one” from among six color chips they did so with success such that memory for this new word was retained by the children over a period of at least a week. Since this pioneering study, FM has been studied extensively and has been described as critically supporting at least the initial stages of language development and the rapid acquisition of vocabulary in very young children [16–19]. FM differs from explicit encoding on several dimensions, including the following. (1) It involves incidental rather than intentional learning, there is no reference for a later test and no effort is made to memorize the new associates. (2) Associations are actively discovered rather than explicitly identified. Disjunctive syllogism, a cognitive reasoning process of eliminating a familiar item before inducing the association between the unfamiliar item and the novel label (“A or B, Not A, Therefore B”), is believed by some to support this process [15, 20]. (3) New information is learned in the context of old information that supports the discovery of the associative relationship and potentially its rapid integration into existing knowledge structures [21, 22]. (4) The new information does not overlap with previous associations, avoiding forgetting through neocortical catastrophic interference [13].

Although primarily investigated in children, it has been suggested that FM serves as a general learning mechanism, not solely dedicated for word learning and as such should be accessible to adults [23, 24]. Indeed, FM has been found to also be available in adulthood [12, 14, 20, 24, 25]. Moreover, this mechanism has been found to exist in other mammals [26, 27] and even birds [28], suggesting it is more than a juvenile language learning apparatus.

Surprisingly, despite its centrality for human knowledge acquisition, there have been very few empirical studies of the cognitive neuroscience of FM [29]. Some theories have postulated that FM is supported by the same neurocognitive systems that support* declarative memory*. Thus, it has been suggested that as in adult declarative learning, only initial acquisition through FM is MTL-dependent [30–33]. Later conceptual rehearsal and storage of word meanings depend on neocortical regions that support gradual and slow storage of context-free information. Furthermore, declarative/procedural distinctions have been suggested similar to the memory systems approach. According to these models, the acquisition of a mental lexicon depends on declarative memory and the MTL, whereas grammar involves procedural memory and is supported by the prefrontal cortex and basal ganglia.

However, the findings showing that FM efficiently functions in very young children in whom episodic memory and the hippocampal system are not yet fully developed [34, 35] led us to hypothesize that FM may be a learning mechanism that is independent of the MTL memory system. To test this, in the first empirical study on this topic, we studied adult amnesiacs with extensive damage to the hippocampus using an adapted FM paradigm [12]. On each incidental learning FM trial in Sharon et al.'s study participants saw two items, one familiar and one unfamiliar (e.g., a dog and a Tenrec) and had to answer a perceptual question about one of them (e.g., “Does the Tenrec have quills?”). Each unfamiliar item was presented twice, each time with a different familiar lure. A surprise forced choice memory recognition test for the unfamiliar items was administered after 10 minutes and again after a week. Patients performed as well as controls on associative recognition both after 10 minutes and after one week demonstrating learning and retaining the learning of arbitrary associations after only two short exposures to the picture-label pairs. By contrast, patients were markedly impaired compared with controls on a matched explicit encoding (EE) task. Importantly, controls performed much better on EE than FM so that task difficulty or depth of processing cannot account for the data. Moreover, preliminary data suggested that the information acquired was declarative in nature; that is, memories were reportable (i.e., conscious) and could be used flexibly, two defining features of declarative memory [36–39]. These data suggest that FM learning might enable rapid acquisition of arbitrary, declarative, and semantic information independently of episodic memory and the hippocampus (see also [13] but see [14, 15] for conflicting findings which we discuss later on). Data from two patients who had extensive anterior temporal lobe (ATL) and who failed to learn associations through FM provide first clues as to the neocortical substrates that may support FM [12]. More recently, we tested short-term (30 minutes) and long-term (24 hours) retrieval of associations acquired either through FM or through EE using fMRI in healthy controls [40]. Connectivity analysis revealed that both short- and long-term items studies through FM drove ATL-centered networks coupled with posterior lateral cortical regions, with little evidence of overnight systems changes. By contrast, there were widespread time-dependent changes in networks supporting retrieval of EE items, driven by VMPFC-hippocampal interactions, in line with our predictions. However, contrary to our predictions, hippocampal activity was also part of some of the FM networks, suggesting that, whether or not the hippocampus is needed for this kind of learning, healthy controls under high levels of interference [13] may recruit the hippocampus during FM (cf. [25]). Either way, these data suggest that, contrary to canonical models of declarative memory, the neocortex may in fact be capable of the rapid plasticity that is required for the acquisition of explicit arbitrary associations and that the ATL is critical for this learning. These surprising findings could have important implications for our conception of neocortical plasticity.

In the present study we continue to investigate the neuroanatomical basis of FM learning using fMRI and multivoxel pattern analysis (MVPA) techniques. We study healthy individuals for two reasons. Data from focal lesion patients can indicate which structures are critical for a particular brain function but do not tell us what other networks might be involved in supporting that function. In addition, brain lesions might change the manner in which an individual performs a task. Thus, investigating large-scale networks in healthy brains while they learn associations through FM is important for understanding the functional neuroanatomy of this early learning mechanism in the adult healthy brain.

Two groups of healthy adults participated in an fMRI study, using the subsequent memory design [41]. In the magnet, participants either incidentally encoded picture-label associations through FM or were explicitly instructed to remember these associations (EE). Memory for the associations was tested outside the magnet after 15 minutes and after a week. Memory performance outside the magnet was then used to investigate the differences in neural networks associated with successful and unsuccessful encoding in FM and EE, respectively. Note that investigating within-condition subsequent memory effects alleviates some of the problems associated with the unavoidable differences between the EE and FM conditions. These differences, such as the incidental nature of encoding, active discovery of the association, and the semantic context within which items appear, are inherent to FM but are characteristic to both successful and unsuccessful encoding of FM associates. Discovering the brain structures that predict FM-induced successful memory would reveal its possible promotion of rapid neocortical learning.

A growing number of studies have demonstrated that machine-learning techniques can be used to extract new information from the neuroimaging data [42–47]. Within the declarative memory field, these techniques have been used to decode specific memories [48] and subsequent memory success [49] and to characterize the information used by participants when they make source memory judgments [50]. The MVPA's advantage in this study is its superior sensitivity to cognitive states and relating brain activity to behavior on a trial-by-trial basis. We investigated large-scale networks using multivariate analysis methods to address the following questions. (1) Can successful versus unsuccessful encoding in FM and EE, respectively, be distinguished from BOLD response? (2) What is the relative contribution of the hippocampus and ATL to classification success in FM and EE, respectively? (3) What other neural structures interact with the hippocampus and the ATL to allow memory for associations in FM and EE, respectively?

To address Question 1 we use* brain decoding*. Brain decoding refers to decoding stimuli, mental states, behaviors, and other variables of interest from the brain scan data (and thereby showing the data contain information about them) and in our case distinguishing subsequently successful and unsuccessful encoding events.  Question 2 involves a mixture of brain decoding and* brain mapping* approaches investigating how the information is mapped onto the activity patterns in particular brain regions. To that end we investigated the decoding success of imaging data when only voxels from the hippocampal region or only voxels from the ATL were used. Based on our patient data [12], we expected the loss in decoding success when only hippocampal data are used would be smaller for EE than FM. Conversely the loss in decoding success when only ATL data are used would be smaller for FM than for EE.  Question 3 integrates a more conventional brain mapping approach and multivariate analysis that is not limited to our target regions but rather investigates which large-scale networks contribute to each of the encoding conditions. Based on previous studies, we expected the EE condition to involve MTL and midline neocortical structures in the frontal and parietal areas. Conversely, if FM reflects direct semantic acquisition, lateral and anterior temporal neocortical areas should emerge.

## 2. Methods

### 2.1. Participants

Forty healthy volunteers were recruited for the study and were randomly assigned to either the FM or the EE conditions. Fifteen participants were excluded from the final data analysis due to the following reasons: three were excluded due to technical problems (1 participant from the EE version and 2 from FM version) and an additional 4 subjects were excluded due to unreliable imaging data (movement artifacts; 1 participant from the EE version and 3 from FM version). Moreover, eight participants were excluded because their memory score was not significantly higher than chance (1 participant from the EE version and 7 from FM version). Note that, in order to allow for sufficient data to be collected in this study, 62 novel stimuli were used in each condition whereas FM has never been tried with such a large number of stimuli before; most studies use 1-2 associations, and our previous study used 16. In anticipation of this inherent difficulty and based on pilot data, we took the approach of testing participants twice and only included participants scoring above chance across both tests. This minimized the probability of inclusion of guessed items as ones that are remembered and increased our confidence that we only included participants who successfully performed the difficult task. Thus, for the statistical analysis, 25 participants were included: thirteen participants who performed the FM paradigm and twelve who performed the EE paradigm. Of these participants, 15 were males and 10 females; their mean age was 26.64 (SD = 3.41). No significant difference was found in the gender distribution between paradigms [*χ*_(1)_^2^ = 0.03, ns] (7 males and 6 females in the FM task and 7 males and 5 females in the EE task), while a marginal difference was found in the age of the participants [*t*_(23)_ = 2.03, *P* = 0.054] in the FM (*M* = 25.38, SD = 2.36) and EE paradigms (*M* = 28.09, SD = 3.93) despite the randomized placement.

### 2.2. Experimental Paradigm

**Figure 1:**
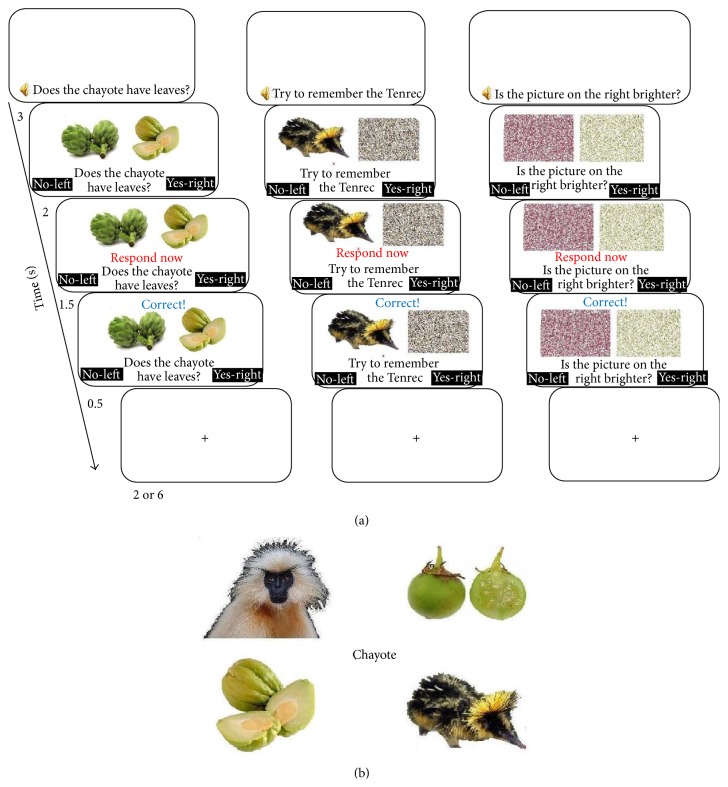
(a) Examples of single trials for FM (left), EE (middle), and baseline (right) stimuli; not to scale. (b) A single retrieval trial (note: EE and FM retrieval trials are identical).

The fMRI versions of the FM and EE paradigms resembled the paradigms used by Sharon et al., 2011 [12] with amnesic patients; however, they were slightly modified to better fit administration in the magnet. The two paradigms were matched so as to share as many visual and motor features as possible (Figure 1(a)) and differed in the unique characteristics of FM. Thus, the FM paradigm in contrast to the EE paradigm included* incidental learning* such that the instructions included no reference to learning or memory processes and* disjunctive syllogism* such that a familiar picture was presented alongside the novel picture. In each paradigm, novel and familiar target trials (either FM or EE trials) were intermixed with baseline trials. Eye-movement experiments [20] have indicated that participants employ an inferential “it is not A, therefore it is B” strategy when learning through FM in similar paradigms and that direct comparisons with known semantic associates are critical for this kind of learning [21]. Novel, familiar and baseline trials were intentionally pseudorandomized across trials. Only the novel target trials were chosen for the subsequent memory analysis. The familiar target trials were inserted to prevent participants from automatically choosing the novel picture as the target without examining the pictures and labels and to add credibility to the instructions that described FM encoding as a perceptual task to make sure that encoding was incidental.

Each trial, whether FM, EE, or baseline, was composed of the following subtrial stages: (1) a question/statement presented both visually and auditorily (3 seconds). (2) The relevant pictures appeared (2 seconds). (3) Participants were given instructions to respond to the question while the pictures remained on screen (1.5 seconds). (4) Subjects received relevant on-screen feedback for their response (0.5 seconds). (5) A red fixation cross was presented for either 2 seconds in half of the events or 6 seconds in the other half. Thus, each event lasted either 9 or 13 s, a mean of 11 s per event.

In the FM task participants were informed they would perform a task designed to examine differences in the way people make perceptual decisions about objects of different levels of familiarity. The stimuli were two pictures of a novel and a familiar animal/fruit/vegetable/flower and the question presented was a perceptual question regarding one of these pictures, the target picture (e.g., “Does the chayote have leaves?”). The target and lure pictures of every trial always differed on the queried dimension such that there was only one possible correct response. The participants were instructed to press either the right button on the response box in order to answer “yes” and the left button if their answer was “no.” No mention was made about a later memory test.

In the EE trials, one picture, either novel or familiar, was presented alongside a scrambled picture and participants were explicitly instructed to remember the item for a later test (e.g., “Try to remember the Tenrec”). Because the FM task involves a visual search and a motor response to a question regarding the stimuli presented, in order to equate the two tasks the participants in the EE task were also requested to look for an x on the screen and, as in the FM paradigm, to press the right (to answer “yes,” if they noticed the x) or left (to answer “no” if they did not see an x) response buttons on the response box in order to answer the question. Finally, in the baseline trials, the participants were presented with two scrambled pictures (the original pictures from the FM paradigms were scrambled) and were asked “Is the picture on the right brighter?” Again, participants were instructed to answer using the response box similarly to the FM and EE trials.

A total of 124 events were designed for each experiment, whether FM or EE. These events were administered in 3 runs such that the first two runs included 40 events and lasted 8 minutes and 2 seconds each and the last run included 44 events and lasted 8 minutes and 52 seconds. The events were organized in 5 sequences of 8 events in the first two runs and an additional sequence of 4 events in the third run. The events were pseudorandomly assigned such that each sequence of 8 events contained 4 novel FM/EE target trials, 2 familiar FM/EE target trials and 2 base line trials (accordingly, the last sequence of 4 events in the 3rd run contained 2 novel FM/EE target trials, 1 familiar FM/EE target trial and 1 base line trial). Every run began with 12 seconds of a presentation of either a reminder of the instructions (on the first run) or a blank screen (the second and third runs). The images acquired during these 12 seconds were intended to allow global image intensity to reach equilibrium, and they were later excluded from data analysis. Between every 8 events a blank screen appeared for duration of 6 seconds. Memory for associations between novel target pictures and novel labels was tested outside the scanner, as described below, using a 4-alternative forced choice recognition in which the label appeared in the center and four pictures around them.

The target picture in the test was one of the novel target pictures presented in the experiment, while the lures were themselves novel pictures presented as targets in other trials in the experiment. The recognition test was designed such so as not to allow a familiarity effect to account for the findings. Participants had to select the correct picture to go with the label (Figure 1(b)). This memory test was administered twice, once about 15 minutes after exiting the magnet and a second time after a week. We only included participants scoring above chance on the first recognition test and above chance across both tests, as tested by the binomial probability to remember the same stimuli in two consecutive tests (binomial test, *P* value used for cutoff 0.05). For the final analyses, responses from the first recognition memory test were used. Items reported by the participants to have been previously familiar (as tested by a yes/no question for each item while items marked as familiar required indicating the name of the item) were omitted from the analysis but this was exceedingly rare.

### 2.3. fMRI Procedure

Imaging was performed on a GE 3T Signa HDx MR system with an 8-channel head coil located at the Wohl Institute for Advanced Imaging in Tel Aviv Sourasky Medical center. The scanning session included T1-weighted anatomical 3D sequence spoiled gradient (SPGR) echo sequences (TR = 9.14 ms, TE = 3.6 ms, flip angle = 13°) obtained with high-resolution 1 mm thick contiguous slices and a 256 × 256 matrix. In addition T2^*∗*^-weighted functional axial images (TR = 2000 ms, TE = 40 ms, flip angle = 90°) were acquired in 32 contiguous slices aligned parallel to the AC-PC plane, of 5 mm thickness with no interslice gap, a field of view of 20 cm, and a 64 × 64 acquisition matrix. The functional images covered the whole cerebrum and yielded 3 × 3 × 5 mm voxels. The images were acquired in 3 runs. In the first 2 runs, 241 images were acquired during each run and in the third run, 266 images were acquired.

### 2.4. fMRI Data Processing

Data were preprocessed using SPM5 (http://www.fil.ion.ucl.ac.uk/spm). The functional images were corrected for differences in slice acquisition timing by resampling all slices in time to match the middle slice. This was followed by a realignment of the time series of images to the first image of the run performed after acquisition of the anatomical image. The data were then spatially normalized to MNI space, detrended, and scaled into the same range. The scaling was done by performing a runwise normalization and computing standard deviation and mean for *z*-scoring based on the volumes corresponding to baseline periods in the experiment.

### 2.5. Multivoxel Pattern Analysis

The multivoxel pattern analysis was used in this study for both brain decoding and brain mapping, taking the advantage of the information contained in the activity patterns across the entire brain volume, from multiple voxels [51].

#### 2.5.1. Brain Decoding (Questions 1 and 2)

For brain decoding, a two-class classification was used. Our features were voxels and the classes were the subsequent memory performance of the participant in the different encoding paradigms. Thus, the trained classifier was essentially a model of the relationship between brain activity during the encoding process and the later recognition performance. The classifier's prediction accuracy was used as a measurement for model quality. To answer Questions 1 and 2, we were interested in “recognition success” versus “recognition failure” for both EE and FM. Trial-level classifier data were obtained by selecting volumes that corresponded to the expected peak of the hemodynamic response function after the appearance of the pictures (i.e., corresponding to 6–8 s postassociative stimulus onset). These were labeled according to the subsequent memory test results, either as “recognition success” or as “recognition failure” for either FM or EE encoding conditions. All data sets were counterbalanced, with an equal number of “recognition success” and “recognition failure” points selected randomly for each experimental run. A linear Support Vector Machine [52] was used as an underlying classifier for all conditions. Both within-subject and cross-subject analyses were performed. Classification results were evaluated using 3-fold cross-validation for within-subject scans (according to the number of the experimental runs) and leave-one-out cross-validation for cross-subject experiments. In all analyses, the accuracy of prediction was based only on test data that were completely independent of the training data.

Considering the high dimensionality of input data, the multivariate classification analysis was preceded by feature selection procedure. The purpose of this procedure is to select the “most relevant” voxels (features) from the high-dimensional set of available voxels. Including feature selection procedure into classification process was shown to improve the classification rate (see, e.g., [53]). The feature selection process was performed on training dataset. In order to decide which voxels should be included in the multivariate classification analysis, each voxel was scored separately using criteria of prediction accuracy, based on *F*-value accepted from ANOVA statistics for differences between “recognition success” versus “recognition failure” conditions for this voxel. Then, 100 voxels with the highest *F*-values were selected for further classification procedure. This selection criterion was empirically determined taking into account the additional improvement in classification accuracy and the cost in computational resources. Increasing this criterion to 500 resulted in only minimal improvement in classification, and from 500 to 1000 there was no improvement at all. A process of feature selection was separately performed, anew for each classification experiment in *n*-fold cross-validation scheme. In all cases, only the training portion of the data was used for this process.

The prediction accuracy was evaluated for both within-subject and cross-subject cases for each encoding condition (EE, FM), using the optimal spatial and temporal aspects of the input data. In the within-subject case, the accuracy value was produced for each participant individually, and then the average accuracy was calculated. In the cross-subject case, the accuracy was produced on the union of all participants' datasets, using the leave-one-out cross-validation method. The accuracy was calculated as an average over all cross-validation folds.

To test the individual contributions of the hippocampus and the ATL to classification in each condition, we replicated the classification procedures described above using either only the data from hippocampal or ATL (BA38/BA21) voxels. Both hippocampal and ATL ROI analyses were carried out using anatomical templates constructed from the WFU Pickatlas 2.5.2 [54]. As a control region we examined the same for the putamen, which was not expected to be differentially involved in FM or EE encoding.

#### 2.5.2. Brain Mapping (Question 3)

Unlike the contrasts classifications, discovering the brain areas associated with each paradigm, FM or EE (Question 3), requires constructing brain maps. In machine-learning terms, brain mapping is a process of highlighting voxels contributing most strongly and reliably to the classifier's success. It may be achieved by determining which voxels are being selected by a classifier and also how their classification weights affect the classifier prediction. The major issue with this straightforward approach is that a group of voxels appearing in a conjunction of all cross-validation fold sets is relatively small (as a result of the initial information redundancy) and cannot be used as a completely reliable source for brain mapping. Information-based functional brain mapping is one method [47] towards overcoming this limitation. These techniques, often referred to as “searchlight classifiers,” work by going voxel by voxel and judging by a multivariate method to what degree the information is in a localized neighborhood of the voxel. We used a relatively large neighborhood of 4-voxel radius (257 voxels) in the experiments, which is still smaller than the volume of subcortical structures that could be of importance (hippocampus, thalamus, and caudate) but is large enough to benefit from the power of multivariate analyses. Smaller radii of 2 and 3 voxels produced spatially very similar information maps but inferior classification performance, suggesting that informative voxels were relatively evenly distributed across the searchlight neighborhoods we report. This is also indicated by the distribution of voxels selected by the feature selection procedure for the whole brain classification. The information checking can be done in different ways; in this paper, we used the same kind of classifier as in our previous experiments (SVM with a linear kernel) but with the data vector projected only onto the searchlight neighborhood. Thus in these brain maps the highlighting color strength reflects the accuracy rate. This rate was used for judging the neighborhood contribution to the differentiation between two experimental conditions, with neighborhoods performing significantly better than a chance level included into the maps.

The software used for these experiments was developed using Python programming language and based on pyMVPA library [55].

## 3. Results

Overall, participants in the FM paradigm answered the yes/no perceptual question correctly, thus correctly inferring that the novel target pictures were the referents of the novel labels, in 85.48% (SD = 6.34%) of the trials. Because in a previous pilot study no difference was found between analysis with or without consideration of the erroneous study trials, items were analyzed irrespective of selection accuracy; the determining factor was subsequent memory of the item-label association outside the MRI. In the EE paradigm, participants correctly identified the x in 95.77% (SD = 3.33%) of the trials; however, this manipulation was intended to equate the EE paradigm to the FM one in terms of visual search, simple perceptual decision making, and motor response and is not hypothesized to be relevant for subsequent memory for the picture-label association. Note that despite the FM task appearing to induce “deeper” encoding, in healthy controls the EE task invariably leads to better memory performance (see below).

### 3.1. Memory Performance

Participants in the FM task recognized a mean of 35.28% (SD = 8.25%) of the associations between novel labels and novel pictures. Participants in the EE task recognized a mean of 43.67% (SD = 8.66%) of the associations. Performance in both tasks was found to be significantly higher than chance (25%) [*t*_(12)_ = 4.50, *P* < 0.001; *t*_(11)_ = 7.47, *P* < 0.001 in the FM and EE tasks, respectively] and a significant difference was found between tasks [*t*_(23)_ = 2.48, *P* < 0.05]. The memory advantage of EE over FM replicates our previous findings in healthy controls [12–14, 25]. Note that the initial advantage was greater and was reduced due to nonproportionate exclusion of participants with chance performance or lower. Reports of preexperimental familiarity of any kind within the included participants were quite rare (average of 0.61 items out of 62; median = 0; range 0–4). These are similar to rates we have in other studies. We did not remove items that included a reportedly familiar lure. However, given the rarity of these items, their influence on performance rates is most likely negligible. Note that this procedure only addresses item familiarity available to explicit report by participants. It does not rule out preconscious familiarity that could bias learning. We hope to address this issue in future studies using implicit measures of familiarity, but for now it remains a potential confound.

### 3.2. fMRI results

**Question 1 (classification of subsequent memory effects in FM and EE).**

**Figure 2:**
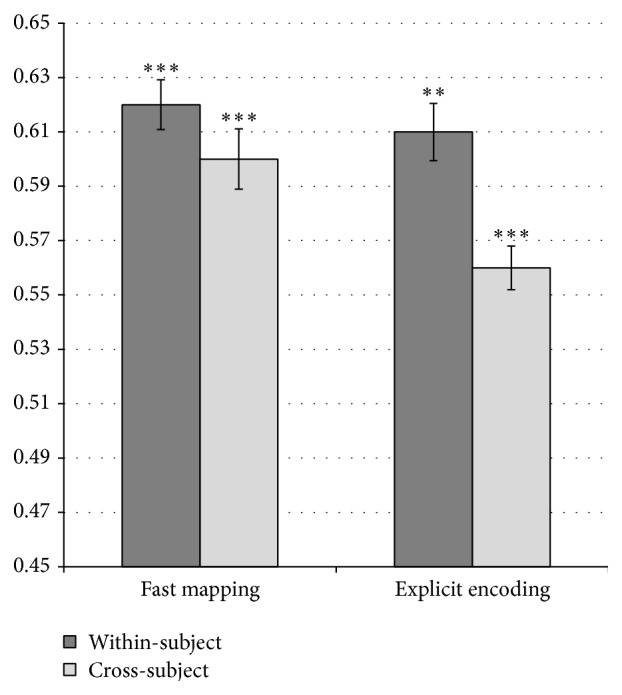
Classification accuracy for within-subject and cross-subject validation procedures. Error bars represent standard error of the mean. ^*∗∗∗*^ *P* < 0.001  ^*∗∗*^ *P* < 0.01, permutation test.

The classification results of the experiment are depicted in Figure 2. They indicate the classification accuracy for within-subject and cross-subject analysis methods. For within-subject method, a mean value of subjects' classification accuracy is reported, with standard error of the mean calculated across subjects. For the cross-subject method, a mean value of leave-one-out cross-validation is reported, with standard error of the mean calculated between different folds.

The classification results indicated that subsequent recognition success could be classified by the SVM for both FM and EE with an accuracy value significantly above chance, as determined by a permutation test implemented in pyMVPA (all *P*'s < 0.01). Dataset-wise label permutation scheme was used for permutation tests. Both training and testing set labels were permuted; however, only the training portion of data was actually relabeled. For within-participant validation, 10000 permutations were used to build the distribution. For cross-participant validation, 1000 permutations were used to build the distribution. Within-participants data comprised of 3 runs. Cross-participant data comprised of 3 runs × 13 participants = 39 runs for FM and 3 runs × 12 participants = 36 runs for EE.

These results provide evidence that information about the future mnemonic outcome of a currently experienced association, whether encoding is incidental through FM or intentional through EE, is present in the neural representations and can be robustly extracted from the BOLD data. Repeated measures ANOVA analysis revealed that validation procedure (within-subject versus cross-subject) significantly differed with respect to classification success (*F*(1,22) = 16.76, *P* < 0.001): within-subject validation produced better classification accuracy (mean difference = 0.058, *P* < 0.001) but there was no feature selection by validation interaction. There was a significant effect of encoding type (FM versus EE) on classification success (*F*(1,22) = 13.07, *P* < 0.005) reflecting better classification accuracy for subsequent memory following FM encoding than EE encoding (mean difference = 0.042, *P* < 0.005). There was no validation procedure by encoding interaction (*P* > 0.05).

**Figure 3:**
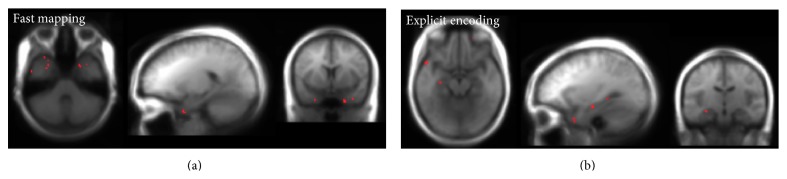
Importance maps resulting from the feature selection procedure of fast mapping (a) and explicit encoding (b).

An examination of the importance maps (Figure 3) of the voxels that were chosen by the feature selection for the whole brain analyses revealed that many of the voxels selected for FM classification reside within the ATL. Other regions were posterior inferior occipitotemporal cortex and parietal lobe. None were in the hippocampus. For the EE the most prominent distribution of selected voxels was within the lateral temporal cortex and the hippocampus.

To better appreciate the performance of the classifier across conditions, Table 1 presents the confusion matrices for the FM and EE classifier outcomes. The True Positive Accuracy calculated as the true positive (tp) divided by the sum of tp and false positive (fp) for the FM classier was 0.59 and for the EE 0.56. The Harmonic Mean of Precision and Sensitivity (or *F*-score calculated as 2*∗*tp divided by the sum of 2*∗*tp, fp and false negative (fn)) for the FM was 0.58 and for the EE 0.56.

**Question 2 (classification success, region of interest (ROI) analysis).**

Given the classification success of the models when features from whole brain activity were used, we were also interested to see whether the hippocampus and ATL had differential contributions for classification of EE and FM, respectively, as would be predicted from our patient data. For this purpose, the classification procedure was repeated for various brain regions including (i) the entire brain, (ii) the hippocampus only, (iii) the ATL only, and (iv) the putamen only. The putamen was selected as a control area for random prediction accuracy with a size comparable to the size of the hippocampus and not expected to play a central role in either FM or EE. Because results showed similar patterns for the two kinds of validation methods, only the results from the cross-subject validation are reported.

**Figure 4:**
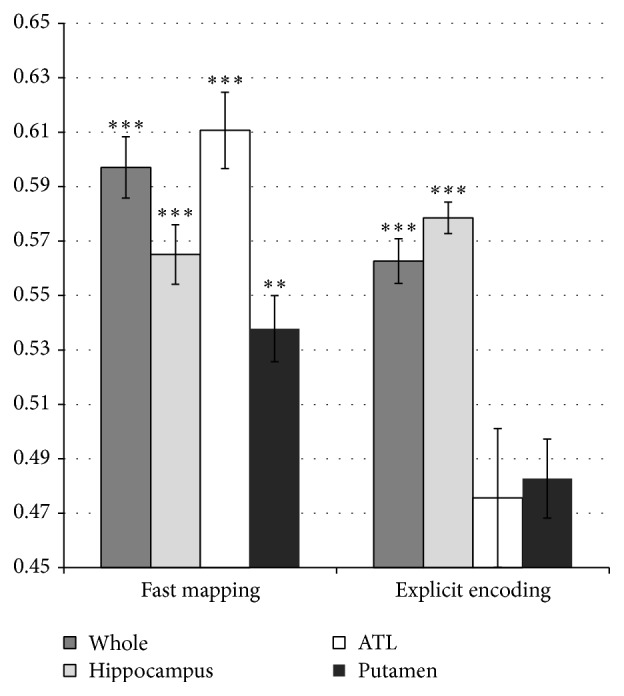
Classification accuracy for cross-subject validation by ROI. Error bars represent standard errors of the mean. ^*∗∗∗*^ *P* < 0.001  ^*∗∗*^ *P* < 0.01, permutation test.

We then conducted a repeated measures ANOVA analysis to examine the effects of ROI for the two groups (Figure 4). Because the sphericity assumption was not fulfilled (*χ*_(2)_^2^ = 15.16, *P* < 0.01) Greenhouse-Geisser corrections were used. Classification accuracy significantly differed across the different ROIs (*F*(1.33, 30.71) = 4.69, *P* < 0.05), and there was an overall group difference in classification accuracy (*F*(1,23) = 13.64, *P* > 0.001), reflecting the better classification of the FM subsequent memory effect. Interestingly, there was a significant ROI by group interaction (*F*(1.33, 30.71) = 18.12, *P* < 0.001). Planned contrasts revealed that this interaction was because (i) classification based on hippocampus alone led to a greater reduction in classification accuracy following FM versus EE (*F*(1,23) = 11.29, *P* < 0.01) and (ii) classification based on ATL alone led to the reverse pattern with greater reduction in classification accuracy following EE versus FM (*F*(1,23) = 13.57, *P* < 0.001). (iii) As predicted, there was no ROI by condition interaction for the Putamen (*F*(1,23) = 0.69, *P* > 0.1), although contrary to our prediction Putamen activity led to above-chance classification in the FM condition.

Our predictions regarding the respective contributions of the ATL and the hippocampus to encoding through FM and EE were only partially fulfilled. As we hypothesized, activity in the ATL could be used to classify subsequent memory following FM just as well as activity in the rest of the brain, whereas using the ATL alone for prediction of EE subsequent memory effect resulted in significant classification decrease and chance performance of the classifier. In addition, using the hippocampus for classification resulted in significant decrease in classification efficiency for FM and improved classification performance for EE. However, even for FM activity in the hippocampus provided sufficient information for above-chance classification. This was unexpected given our previous findings with hippocampal amnesics, but consistent with our [13, 40] and others' [25] prior studies with healthy controls. It may be that in healthy individuals the intact hippocampi automatically participate in encoding of environmental stimuli. The use of a very long list of stimuli may also be a contributing factor for hippocampal involvement in FM in the present study due to increased interference. Nonetheless, the basic pattern of relative hippocampal/ATL involvement in FM and EE was consistent with the idea that FM enables more rapid and direct neocortical integration [13, 21, 40].

**Question 3 (Brain Activation Mapping).**

Next we sought to examine which large-scale networks beyond the hippocampus and ATL contribute to prediction of subsequent memory in each of the conditions and whether these networks significantly differ from one another. The results from the region specific classification analyses indicate that, in all likelihood, subsequent memory effects of FM and EE rely on both overlapping and distinct neural networks.

**Figure 5:**
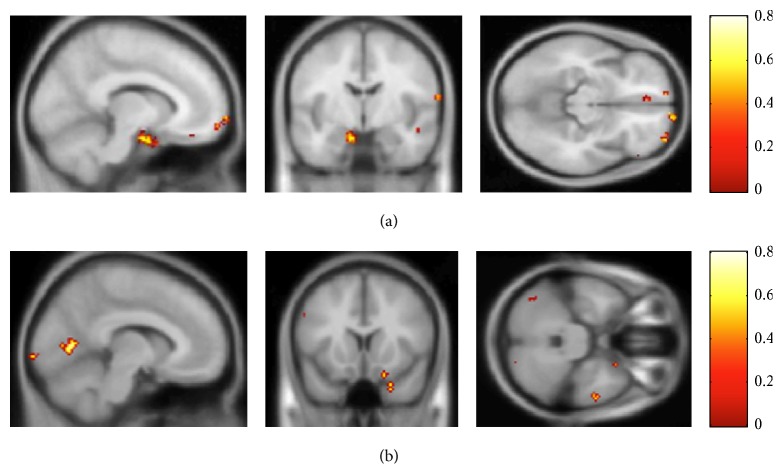
Searchlight results for the explicit encoding condition (a) and fast mapping condition (b) across participants.

The results presented below were obtained with a “searchlight” algorithm (see Section 2) representing a two-way classification between successful versus failed recognition conditions. The overall pattern is that the FM model displays a more distributed pattern that involves many more brain regions than the EE model (Tables 2 and 3 and Figure 5). Examining the specific patterns, it is apparent that EE is associated with regions in the medial temporal lobe (MTL) including the hippocampus, supporting the finding from the classification ROI analysis. EE is also associated with extensive bilateral ventral medial prefrontal cortices (VMPFC), right lateral prefrontal cortex (dorsal and ventral), anterior cingulate, and right posterior lateral temporal neocortex (Table 2, Figure 5(a)). These are regions typically seen in studies of subsequent memory effects in declarative memory.

By contrast, FM was associated with bilateral anterior temporal lobe (ATL; BA38), again confirming the classification ROI analysis. FM memory success was also prominently associated with more posterior lateral and inferior temporal neocortical regions and posterior inferior occipital cortices. Frontal lobe involvement included orbitofrontal, dorsolateral, and ventrolateral PFC, but no VMPFC (Table 3, Figure 5(b)).

This pattern converges with the importance maps identified by the feature selection for the whole brain classification analysis and with the ROI analyses results but extends it to additional structures whose activity contains information about subsequent memory. The extent to which this pattern is driven by overall voxelwise levels of activity that is typically studied using univariate analyses could not be formally assessed because these analyses lacked sufficient statistical power for a subsequent memory test of the kind performed here [56].

## 4. Discussion

The present paper investigated subsequent memory effects for semantic encoding through FM contrasted with standard intentional encoding in healthy individuals. Previous studies with amnesic patients have indicated that FM encoding allows for hippocampal-independent acquisition of novel arbitrary associations and that a neocortical structure that may be crucial for this type of learning is the ATL [12, 13]. Here we demonstrate that it is possible to classify with great accuracy encoding-related neural states that lead to subsequent successful associative recognition from those associated with failed recognition. Moreover, in partial agreement with the patient data, the hippocampus contained relatively more information about subsequent memory status for the EE condition, although subsequent memory accuracy following FM could also be classified from hippocampal activity. By contrast, the ATL contained more subsequent memory information for FM than the hippocampus, and EE subsequent memory performance could not be classified based on ATL activity at all. Finally a searchlight algorithm approach demonstrated very distinct networks associated with successful compared with unsuccessful memory encoding process. FM successful encoding was primarily related to ATL, inferior-posterior lateral temporal and occipital neocortex and ventrolateral prefrontal cortex. By contrast, EE successful encoding was related to MTL activity (including the hippocampus), midline structures of the prefrontal and parietal lobes, and lateral temporal/temporoparietal junction cortex, in line with previous neuroimaging studies of episodic encoding.

### 4.1. The ATL and Acquisition of New Semantic Associations

Much of our semantic knowledge, including word meanings and conceptual knowledge, is acquired during childhood and forms the basis for later memory. The developmental literature points to fast mapping as a key mechanism in this process, supporting the initial stages of concept formation [24, 57] and the lexicon [16–19]. However, the fundamental functional neuroanatomy of this central mechanism remained unknown. We suspected that learning through FM involves rapid and direct changes to neocortical structures, as also suggested by a handful of neurophysiological studies [29]. These include structures that are implicated in semantic memory such as the lateral and anterior temporal lobes (ATL), posterior temporal neocortex, and ventrolateral prefrontal cortex (PFC) [36, 58–62]. The results of testing two patients with left ATL damage who failed to learn through FM lent initial support to this hypothesis [12]. A recent imaging study during retrieval also suggested the ATL serves as a hub for posterior neocortical networks that represent associations generated by FM almost immediately; these networks were only weakly present during retrieval of associations generated by EE, and only after a night's sleep, presumably following network reorganization [40]. Our current results strongly support these initial observations. The region of interest classification analysis demonstrated that a high proportion of the information about subsequent memory performance during FM encoding could be exclusively derived from voxels in the ATL. By comparison, EE classification success was reduced to chance when only the ATL was used. Moreover, a whole brain searchlight algorithm with no* a priori* hypothesis about the ATL further demonstrated its important role as part of a network that predicts the formation of associative memory during FM. Bilateral ATL cortical areas appeared as part of the extensive neocortical network that supports FM, while ATL did not appear in the EE searchlight analysis, suggesting it does not play an immediate central role during such learning.

The neural representation of semantics involves broadly distributed circuits that reflect distinct conceptual categories and their associated properties, such as perceptual, motor, functional, and affective features [60, 62–64]. According to “semantic hub” models [58, 60, 65] an amodal, domain-general hub is required to bind together these discrete property regions, a function served by the ATL. According to the semantic hub model, the activity in the ATL observed in the present study reflects the binding together of properties represented in posterior neocortex to allow fine-grained distinctions between perceptually similar stimuli, a type of neocortical pattern separation. Subsequent successful associative recognition following FM may depend on the creation of efficient ATL-mediated amodal representations of coactivated areas in posterior neocortex. A similar model suggests that the ATL serves as an intermediary “convergence zone” system for triggering word form retrieval given conceptual knowledge, and vice versa for unique entities [66, 67]. Retrieval of novel items (e.g., the word Tenrec) after a single encounter is similar to retrieval of unique entities, that is, specific and precise knowledge including proper names, for stimuli that are concrete and unique [68]. ATL activity in the present study would thus reflect the formation of an index that associates a novel word form with novel conceptual knowledge.

If, as we suspect, the ATL is critical for direct neocortical acquisition of names of unfamiliar animals, fruit, flowers, and so forth, then it would be compatible with both the “semantic hub” model and the “convergence zone”' account. Either way, it would be expected that, in addition to the ATL activity, posterior temporal and occipital neocortical regions would be coactivated when such associations are being laid down, as described below.

### 4.2. Posterior Temporal, Subcortical, and Prefrontal Contributions during Semantic Learning through Fast Mapping

In addition to the ATL that proved important for FM in both the classification and the searchlight analyses, FM successful memory classification also relied on posterior and frontal neocortical regions. These patterns appeared to be more distributed and widespread than the ones predictive of EE successful recognition and to involve more lateral and inferior neocortical regions. We propose that this widespread neocortical activity may be a signature of direct semantic acquisition. The activated neocortical networks identified by the searchlight algorithm may reflect activation of existing knowledge structures that support acquisition of novel associations. FM successful encoding depended on extensive cortical regions in the middle occipital gyrus and in the fusiform gyrus bilaterally. These areas constitute part of the visual ventral stream and have been described in previous studies as correlated with different stages of object recognition [69–71]. According to Zannino et al. [72], ventral occipitotemporal activations are the most consistent finding in neuroimaging studies of semantic processing. Its activation during semantic tasks has been a basis for the theory that claims that semantic knowledge is mediated in the brain by the same areas by which it is encoded [64]. Interestingly, in addition to structures associated with the visual stream, the searchlight algorithm also identified extensive activation in secondary and tertiary auditory cortical regions. Caudal area 22 in the left superior temporal gyrus is considered to be a major component of Wernicke's area and support processing of lexical semantics [73]. It is important to note that while posterior regions are likely where the actual representations of the features that make up novel lexical semantic entries reside, by no means do the patterns picked up by the classifier reflect specific item identities. Our task and the spatial and temporal specificity of fMRI could not possibly capture that information. Moreover, such information even if captured, could not be diagnostic of the broad categories of “remembered” versus “forgotten.” Instead, what the classifier might tap onto is a pattern within higher-level unimodal associative cortices that reflects successful generation of unique codes in lower level perceptual regions. Thus, successful encoding through FM is predicted by coactivation of modality-specific perceptual representations of items and their associated auditory information, coupled with activation of the ATL amodal hub in which higher-level semantic knowledge is represented [60]. Object-label associates that engage these regions to a greater extent are more likely to be effectively processed and for relations among their features to be created. Subsequently these associations are more likely to be later retrieved from memory. Some experimental evidence that neocortically represented features of semantic representations acquired through FM may significantly overlap was recently presented by Merhav et al. [13]. They demonstrated that, in both healthy controls and patients with amnesia using AB-AC interference, overlapping paired-associates learned through FM suffer from catastrophic interference, a hallmark of independent neocortical learning, while associates studied through EE are immune to such interference presumably by virtue of their hippocampal component [13].

These patterns also are consistent with the prominence of linguistic processing in memory through fast mapping. The posterior regions the classifier identified have been implicated in language processing as parts of both the dorsal stream, along the superior longitudinal fasciculus, and the ventral streams, along the inferior frontooccipital and uncinate fasciculi [74]. Successful fast mapping, as implemented in our task, requires both parsing of linguistic information to identify the target stimulus and holistic representations of lexical semantics that are mediated by dorsal and ventral streams, respectively. Previous studies of rapid perceptual learning of word forms have similarly identified changes in perceptual lexical processing associated with dorsal stream regions [75, 76]. By contrast, the combination of lexical semantics and lexical phonology acquired through FM engages both dorsal and ventral streams. The EE task, on the other hand, could be decoded primarily by right-sided lateral cortical regions, medial prefrontal and medial temporal structures that are structures associated with visual episodic memory rather than a linguistic network.

One region that we had not expected to be predictive of successful memory following FM is the putamen, which classified at above chance levels for the FM but not EE conditions. The dorsal and ventral striatum are differentially connected to discrete prefrontal cortical regions in segregated corticostriatal circuits [77]. The putamen plays a critical role within the so-called “motor circuit” while the caudate forms part of the oculomotor, dorsolateral, and ventral/orbital circuits. This is why we assumed it would not play a significant role in vocabulary learning through FM. We were similarly surprised; however, to find putamen was part of functional networks that support retrieval of FM (and not EE) associations in our recent study of retrieval [40], suggesting this is more than a coincidence or some “leakage” of information from other structures. One possibility is that the role of the putamen and in particular the left putamen in language production (initiation and execution of speech [78]) as part of its connectivity with the supplementary motor somehow supports the novel lexical entries acquired through FM. Interestingly the left SMA also features prominently in the searchlight results for the FM, which may be part of the same network.

#### 4.2.1. Fast Mapping and Prior Knowledge

One characteristic of FM that may determine the way new information is acquired is that novel information appears in the context of already known items. FM learning is thought to depend on preexisting categorical or conceptual knowledge [22, 79]. Previous studies of amnesia suggest that anchoring new knowledge onto existing knowledge may allow patients to acquire a new language and lexicon [80] and even patient HM acquired new facts when these were anchored to information he knew [81]. We have suggested that conditions in which the associations are completely novel (i.e., both item and label have never been encountered before) but are clearly embedded within existing conceptual frameworks are conducive for rapid modification of connection between neocortical nodes [12, 13]. This is consistent with much of what we know about prior knowledge effects on new learning [9, 82] and also with some evidence on FM. Recent work using similar stimuli to ours, but nonsense words, demonstrated that encoding through FM leads to immediate lexical integration, as indexed by lexical competition effects on the processing of neighboring existing words [21]. It also led to semantic priming effects after a day, compared to EE that led to stronger declarative memory but no evidence for either lexical or semantic integration [21]. Importantly, both effects required that a competing item be present, suggesting that activation of existing semantic networks is an inherent part of the process of integration of novel associations. It has also been demonstrated that the more transparent the distinction between target items and competing items in the environment is, the more likely the correct mapping would occur and be maintained cross-situationally [83]. Therefore, it appears that variations in levels and richness of knowledge of the competing items affect mapping and learning of new items, such that the more one knows about the competing items the more likely the novel associations can be formed. The activation predictive of later memory of novel items through fast mapping could reflect both the activation of old associations and the integration of new information, which are indistinguishable under this model. Similar processes have been shown to be central in other domains of memory including reconsolidation of conditioned associations and updating of episodic declarative memory [84].

Recently, Tse and colleagues [7, 8] elegantly demonstrated that when novel flavor-location pairs are studied in the context of old well-established schematic flavor-location setting, neocortical consolidation is greatly accelerated in rats. While this is consistent with our claim that the neocortex is capable of rapid consolidation of novel arbitrary associations, we note that initial acquisition in that study was still reliant on the hippocampus. Importantly, our data indicate that the ATL is a critical epicenter for the formation of novel associations through FM when novel associations are embedded in previous conceptual knowledge. Tse and colleagues [7, 8] on the other hand present evidence that the prelimbic cortex, a homolog of the human medial PFC, was key for schema-based hippocampal-dependent rapid consolidation. As we discuss below, the medial PFC appears critical during acquisition and transformation of hippocampally dependent episodic memory into independent neocortical semantic memories [6, 85, 86] particularly when novel memories are embedded in schematic knowledge [9]. This contrasts with FM that depends of prior conceptual knowledge, rather than schema, and in which learning is mediated by the ATL.

### 4.3. Hippocampal-Medial Prefrontal Interplay during Learning through Explicit Encoding

While FM subsequent memory could be primarily classified by activations in ATL, PFC, and widespread posterior and inferior neocortical structures, the EE ROI classification and searchlight results demonstrated a very different pattern. Successful subsequent performance following EE could be predicted quite efficiently based on hippocampal voxels alone. The ATL-only model on the other hand was associated with significantly greater reduction in classification success and in fact led to chance performance of the classifier. That pattern was the reverse of what was observed for the FM, forming a “double dissociation” between the two encoding conditions. This pattern is consistent with the idea that the hippocampus critically supports acquisition of novel associations under standard encoding conditions and replicates numerous other studies of subsequent memory effects in episodic memory tasks [41, 87–89]. The findings from the ROI classification analysis were later confirmed by the searchlight algorithm with no a priori delineation of structures, which also identified the hippocampus and parahippocampal gyrus as key regions in a limited collection of regions that together predicted subsequent memory following intentional encoding.

The other major contributor identified by the searchlight was the medial aspect of the prefrontal cortex bilaterally as well as dorsolateral and ventrolateral PFC. Within the memory literature, ventral medial prefrontal cortex (VMPFC) has mostly been identified as a key structure in retrieval processes of episodic [90, 91] and semantic [62, 92] memory. The VMPFC is intimately connected with limbic and paralimbic declarative memory structures including the hippocampus, parahippocampus, and retrosplenial cortex [93, 94]. Although it has been linked primarily to motivation and reward processing [95, 96], damage to VMPFC can also lead to memory syndromes. Specifically, lesions to that region can lead to confabulation (false memories) that may arise from deficits in automatic memory monitoring (“felt rightness” [97, 98]. “Felt rightness” deficits may reflect failed automatic biasing of relevant posterior neocortical representations and consequent erroneous retrieval of strong (but irrelevant) attractor schematic knowledge [92, 99, 100] cf. [101] for a detailed cognitive model). The idea that VMPFC interacts with posterior neocortical structures also figures prominently in theories that suggest this neocortical structure is key to the process of systems consolidation or reorganization as episodic memories are transformed from being hippocampally bound to being hippocampal-independent [6, 8, 85, 86]. Some have even suggested that when new associations are highly congruent with existing schema, the VMPFC may take over the role of binding together these associations independently of the MTL [9]. Either way, increased activity in medial prefrontal cortex during successful intentional memory encoding may reflect the use of specific encoding strategies and recruitment of attentional and executive resources that together with the MTL provide the substrate that allows for later memory. It may contribute to successful concept interference resolution as our stimulus set involved labels and items that were similar to each other. Associating novel information to existing knowledge at encoding through VMPFC connections may have supported pattern separation performed by the hippocampus and allowed for later accurate associative recognition.

### 4.4. The Hippocampus and Encoding of Associations through FM

The discussion so far highlighted the preferential role of anterior, later, and posterior neocortical regions in encoding associations through FM compared with the hippocampal-VMPFC axis that appear to be central for encoding through EE. However, hippocampal voxels contained enough information to allow above-chance classification in FM in the present study. Moreover, the finding that FM can support learning independently of the hippocampus in patients with amnesia is by no means unanimous [14, 15]. The study by Warren and Duff [15] deviated significantly from standard FM tasks, which may have led to the difference in findings. For example, the participants in that study were provided with explicit memory instructions, while FM typically involves incidental learning. Moreover, items were intensively trained prior to associative learning, possibly interfering with the novel item to novel label mapping. Indeed, it appears that the EE and FM conditions in the Warren and Duff study likely did not tap different encoding processes, as suggested by the finding that control participants performed equally well on the two conditions, failing to show the typical EE advantage reported in this study and others. The study by Smith et al. [14] more closely resembled the studies by Sharon et al. and Merhav et al. It was suggested that specific aspects of task administration affected the memory performance or that FM may support learning in the context of less severe amnesia [14]. Future studies should clarify these questions.

One possibility that may account for the differing results from amnesic patients and the involvement of the hippocampus in FM in our neuroimaging study is that the paradigms we use involve higher levels of interference than typical developmental fast mapping occurring either naturally or in the lab. A typical lab experiment of FM involves very few items that are very distinct and allow for very distinctive interaction with the items. We use much longer lists (sixteen items in the patient studies and over 60 here) of highly similar items. These features may induce hippocampal activity to allow for interference resolution through pattern separation. Smith et al. [14] also suggested that learning through FM might not be robust, and the study by Merhav et al. [13] demonstrating catastrophic interference supports that notion. Coutanche and Thompson-Schill [21] made similar observations with respect to FM's failure to support free recall, which contrasted with its superior ability to integrate new information into existing semantic networks. Within the developmental literature it has been suggested that associations acquired through FM initially have a “hypothesis status” [83, 102] such that if contradictory evidence is encountered, the system “re-sets” and all associations are erased. This erasure susceptible state is presumably maintained until novel meanings are refined and confirmed by the child, to prevent perpetuation of foundational errors [83, 102]. In healthy adults this interference susceptibility may induce automatic hippocampal activation that could contribute to FM learning but obviously to a lesser extent and in parallel to the more prominent neocortical structures.

## 5. Conclusion

It appears that although both FM and EE lead to the acquisition of declarative memory as reflected in the postscan recognition performance, they do so by recruiting very distinct neuronal networks that can be efficiently distinguished using the machine-learning classifier SVM. Thus, although healthy individuals possess the neural machinery that should allow for encoding information using the MTL memory system, under conditions that promote FM, they do not rely on the MTL to the same extent. An alternate explanation of this finding is that the FM and EE conditions differ on some perceptual and motor aspects and that the classifier may have picked up on features associated with these differences. We cannot completely rule out that possibility; however, we note that the classification was performed within each condition and that subsequently remembered and subsequently forgotten items did not differ on any of these features, other than on the final outcome of memory. We also note that the regions associated with each condition (i.e., separating successful and unsuccessful subsequent memory within each condition) are consistent with the hypothetical areas of each memory system and our previous patient lesion studies. This suggests the information was extracted from functionally relevant areas to each memory system rather than those that are only activated by peripheral aspects of the task demands. Either way, the finding that learning declarative information can be supported directly by the neocortex is surprising and challenges certain aspects of current theories of FM [30, 32, 103]. It also suggests there are exceptions to current more general theories of declarative memory that preclude the possibility of direct neocortical plasticity [3, 104, 105].

## Figures and Tables

**Table 1:** Confusion matrices for FM and EE.

		Predicted label	
		Remembered	Forgotten
FM			
Real label	Remembered	161	119
	Forgotten	113	167

EE			
Real label	Remembered	173	134
	Forgotten	136	171

**Table 2:** Searchlight results for subsequent memory effects of explicit encoding.

Region			*x*	*y*	*z*
Frontal lobe	Inferior frontal gyrus (47)	R	52	32	−16
	Inferior frontal gyrus (9)	R	48	18	−16
	Medial frontal gyrus (10/9)	L	−12	58	20
	Medial frontal gyrus (10/11)	L	−12	68	−10
	Medial frontal gyrus (10)	L	−12	66	−6
	Orbitofrontal cortex (11)	R	12	66	−24
	Medial frontal gyrus (10/11)	R	14	68	−12
	Medial frontal gyrus (10)	R	10	64	−8
	Precentral gyrus (6)	R	68	−2	16
	Superior frontal gyrus (10)	R	36	62	−12

Parietal lobe	Postcentral gyrus/IPL (2/40)	R	46	−24	30

Temporal lobe	Hippocampus	R	28	−36	−1
	Parahippocampal gyrus (34/28)	L	−10	−6	−20
	Middle temporal gyrus (20/21)	R	64	−12	−14
	Middle temporal gyrus (21)	R	50	−6	−12

Other	Culmen of cerebellum	L	−36	−40	−26
	Anterior cingulate (32)	R	1	38	−4
	Anterior cingulate (32)	L	−6	38	−14
	Caudate (head)	L	−8	8	−4

**Table 3:** Searchlight results for subsequent memory effects of fast mapping.

	Region		*x*	*y*	*z*
Frontal lobe	Inferior frontal gyrus (47)	R	50	28	−10
	Orbitofrontal gyrus (11)	R	10	14	−22
	Orbitofrontal gyrus (11)	R	4	32	−18
	Middle frontal gyrus (9)	L	−54	18	32
	Precentral gyrus (6)	L	−58	0	16
	Precentral gyrus (6)	L	−34	−18	66
	Middle frontal gyrus (6)	R	46	0	58
	Superior frontal gyrus (9)	L	−20	48	32

Occipital lobe	Cuneus (19)	L	−14	−78	38
	Cuneus/precuneus (18/31)	L	−18	−74	22
	Inferior occipital gyrus (19)	L	−40	−76	−4
	Middle occipital gyrus (19)	L	−38	−78	6
	Lingual gyrus (18)	L	−8	−72	2
	Cuneus/lingual gyrus (17/18)	L	−12	−100	−2
	Cuneus/lingual gyrus (17/18)	R	24	−102	−2
	Middle occipital gyrus (18)	L	−28	−92	14
	Middle occipital gyrus (19)	L	−38	−84	10

Parietal lobe	Inferior parietal lobule (40)	L	−36	−54	44
	Postcentral gyrus (40)	R	46	−28	62

Temporal lobe	Fusiform gyrus (37)	L	−40	−62	−10
	Inferior temporal gyrus (20)	R	58	−6	−32
	Middle temporal gyrus (21/22)	L	−66	−48	2
	Middle temporal gyrus (21/22)	R	64	−34	2
	Superior temporal gyrus (38)	R	50	24	−20
	Superior temporal gyrus (38)	L	−36	14	−28
	Superior temporal gyrus (22)	L	−52	−50	16
	Middle temporal gyrus (21)	L	−52	−22	−6
	Middle temporal gyrus (22)	R	62	−34	8

Other	Cerebellar tonsil	L	−44	−68	−36
	Cerebellar tonsil	R	54	−48	−44
	Cerebellar declive	L	−38	−76	−16
